# Strengthening the Self-Assembly of Supramolecular Polymeric Nanotubes in Water via the Introduction of Hydrophobic Moieties

**DOI:** 10.1021/acsmacrolett.4c00759

**Published:** 2025-02-21

**Authors:** Zihe Cheng, Stephen C. L. Hall, Qiao Song, Sébastien Perrier

**Affiliations:** † Department of Chemistry, University of Warwick, Coventry CV4 7AL, U.K.; ‡ ISIS Neutron and Muon Source, Rutherford Appleton Laboratory, Didcot OX11 0QX, U.K.; § Shenzhen Grubbs Institute, Southern University of Science and Technology, Shenzhen 518055, China; ∥ Warwick Medical School, University of Warwick, Coventry CV4 7AL, U.K.; ⊥ Faculty of Pharmacy and Pharmaceutical Sciences, Monash University, Parkville, Victoria 3052, Australia

## Abstract

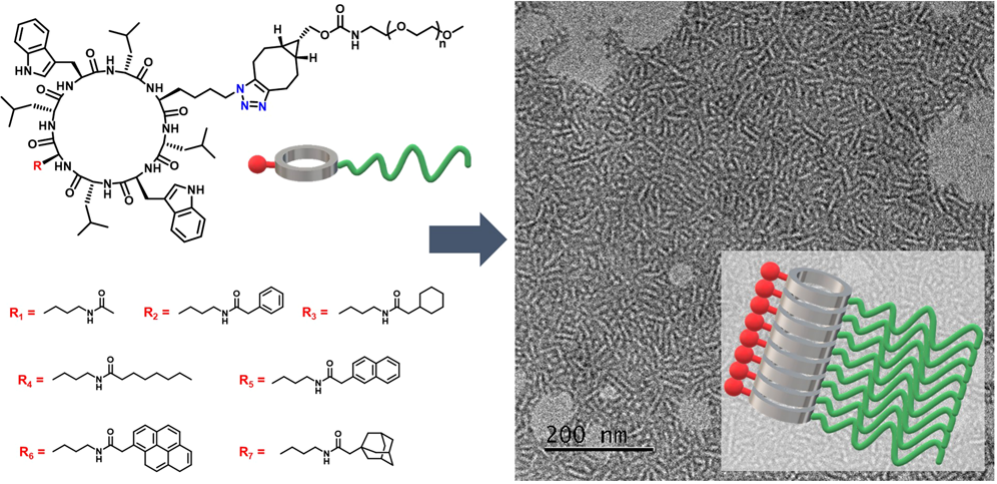

Supramolecular polymeric nanotubes based on the self-assembling cyclic peptide–polymer conjugates are a promising class of materials, showing great potential in various biological applications. Herein, we present a novel strategy to promote nanotube assembly through effectively shielding the cyclic peptides from water, via the introduction of varying hydrophobic groups. As determined by a combination of SANS, TEM, and SLS, hydrophobic interactions, π–π stacking, and multiple hydrogen bonding interactions cooperate in the self-assembly of the cyclic peptide–polymer conjugates, allowing for the construction of supramolecular nanotubes that are longer than expected in water. This approach offers an effective pathway toward the design of organic nanotubes of hundreds of nanometers in water.

## Introduction

Supramolecular chemistry enables the design of highly ordered chemical systems by noncovalent interactions, including hydrogen bonding, metal coordination, π–π interactions and hydrophobic interactions.^1^ Although these interactions are relatively weak (0.1–5 kcal mol^–1^), they play a vital role in fundamental biological processes such as protein folding and the expression and transfer of genetic information.^2^ Hydrogen bonds, which are among the most encountered interactions in supramolecular chemistry, occur between polar X–H bonds and nonbonding electron pairs on atom Y, where X and Y represent a series of electronegative atoms, such as nitrogen, oxygen and fluorine.^2,3^ While a single hydrogen bond is weak, the cumulative effect of multiple hydrogen bonds significantly increases the interaction strength between two molecules and guides the formation of higher-order supramolecular assemblies.^4−8^ Supramolecular interactions in aqueous environments are particularly intriguing for practical applications; however, they represent challenges due to water molecules acting as strong hydrogen bonding competitors, which can disrupt the foundational hydrogen bonding necessary for supramolecular structure formation.^9^ To achieve better control over their architecture, the construction of supramolecular assemblies in water necessitates hydrophobic shielding to mitigate the interference from water molecules.

Among the various nanostructures formed through noncovalent interactions, supramolecular nanotubes assembled by cyclic peptides represent a particularly promising class of materials, first reported by Ghadiri, Granja and co-workers.^10^ These structures are composed of cyclic peptides with an even number of alternating d- and l- amino acids, which stack into nanotubes driven by multiple hydrogen bonding interactions. The unique hollow tubular structures have demonstrated a strong potential for a great range of biological applications, such as transmembrane channels^11−14^ biosensors^15^ and drug delivery.^16^ However, one of the major drawbacks of these materials is their tendency to laterally aggregate, which significantly reduces their solubility in solvents, especially in water. To deal with this issue, conjugating polymers to the periphery of the peptide rings has been shown to prevent lateral aggregation and drastically enhance solubility. In this case, cyclic peptide–polymer conjugates (CPPCs) can self-assemble into polymeric nanotubes with well-defined core–shell structures. Moreover, the properties and functionalities of these assemblies can be effectively tuned by modifying the grafted polymeric chains, thereby broadening their range of applications. To date, a variety of polymers including poly(styrene) (PS), poly(butyl acrylate) (pBA), poly(ethyl oxazoline) (pEtOx), poly(acrylic acid) (pAA), poly(2-nitrobenzyl methacrylate (pNBMA), polyethylene glycol (PEG) have been conjugated onto cyclic peptides to form supramolecular polymeric nanotubes.^17^ While it is widely acknowledged that the dimensions of supramolecular aggregates greatly impact their properties and potential applications, the dynamic nature of the supramolecular driving forces makes it challenging to design supramolecular polymeric nanotubes with tailored dimensions. In general, the length and diameter of these self-assembling nanotubes are affected by the size and topology (linear or brush like) of attached polymer chains, regardless of the polymers. The diameter of polymeric nanotubes tends to increase with the molar mass of the polymers, while the length decreases due to the steric repulsion of bulky polymer chains.^18,19^ In addition, the self-assembly process in solution is affected by the graft density of the attached polymer arms, as well as hydrogen bonding capacity and polarity of the solvent.^20^ Recently, Rho et al.^21^ introduced a secondary hydrophobic interaction by attaching amphiphilic block copolymers to the peptide rings, creating a hydrophobic inner shell which shields the cyclic peptides from water. This approach allowed the formation of longer nanotubes compared to those obtained from cyclic peptide–polymer conjugates based on homopolymers.

However, the requirement for diblock copolymers limits the applicability of this approach. Therefore, there remains a need for a more straightforward strategy to promote nanotube assembly by effectively shielding the peptide core from water.

A similar approach has been employed in the assembly of benzene-1,3,5-tricarboxamides (BTAs), which self-assemble into helical stacks through triple hydrogen bonding between amide groups.^22^ Leenders and co-workers reported the formation of supramolecular fibers in water using BTAs derivatives through introducing hydrophobic aliphatic groups adjacent to the core to provide sufficient shielding for the hydrogen bonds, while hydrophilic tetraethylene glycol groups at the periphery enhanced solubility.^23^ This work highlighted the potential of combining hydrophobic effects and hydrogen bonding interactions for the formation of supramolecular assemblies in water. Furthermore, Klein et al.^24^ revealed that the morphology of polymeric BTA amphiphiles in water was more influenced by the packing parameter than by the directed hydrogen bonds of BTA motifs, due to a strong surface tension. When the length of hydrophobic alkyl spacers was varied, anisotropic structures were observed for a C_12_ alkyl spacer, while spherical structures were observed for C_6_ and C_10_ spacers. To reduce the packing parameter effect of the amphiphilic polymers, the hydrophobic moieties was directly attached to the self-assembling scaffold.

**Scheme 1:**
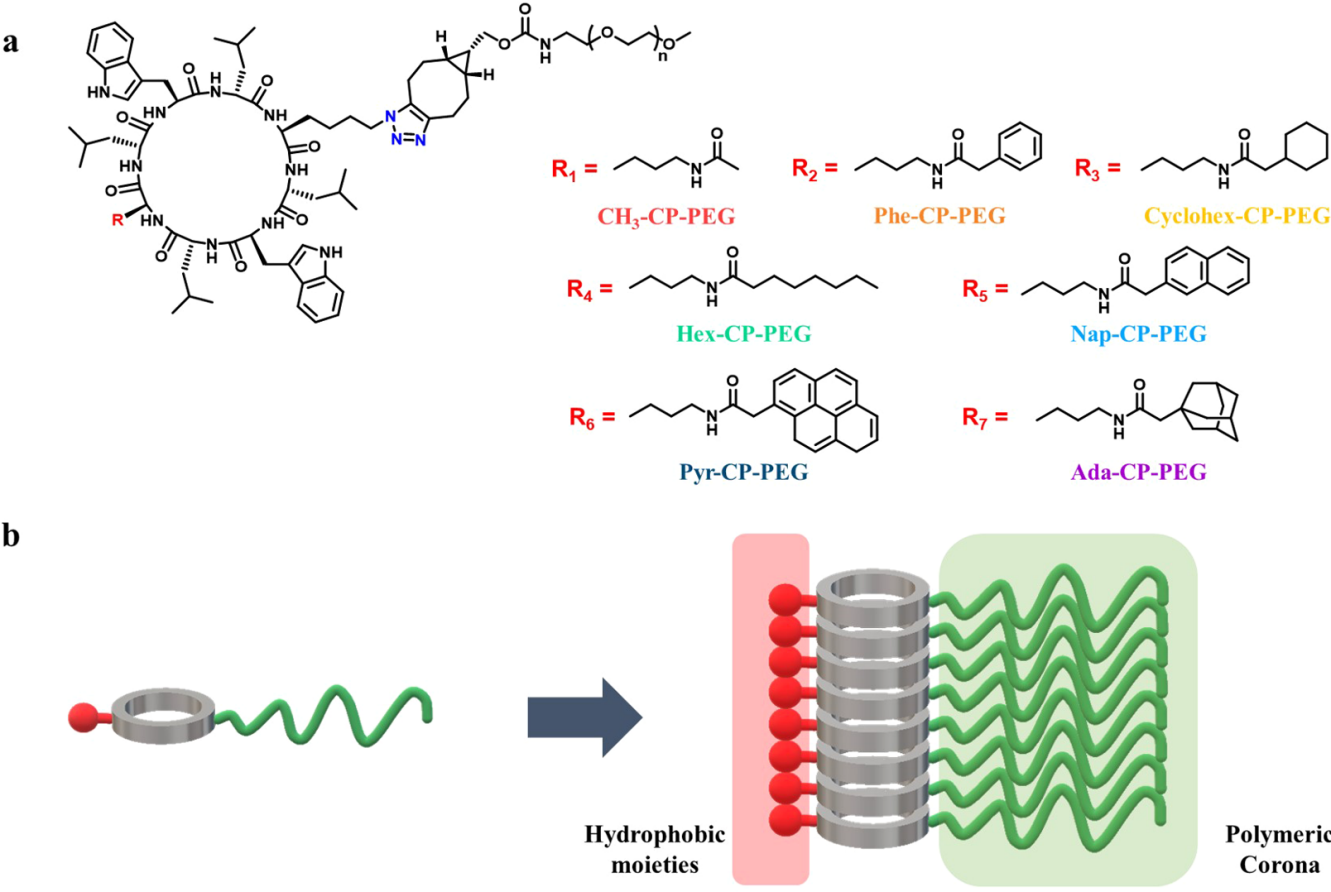
(a) Chemical Structures of the Cyclic Peptide Conjugates Investigated in This Study; (b) Schematic Illustration of the Supramolecular Peptide Nanotube Formed by the Introduction of Hydrophobic Moieties

Herein, we propose a novel approach to regulate the self-assembly of cyclic peptides, where a range of hydrophobic groups are covalently attached to the cyclic peptide in peptide–polymer conjugates. These hydrophobic groups are expected to shield the cyclic peptide cores from water molecules, thus stabilizing the multiple hydrogen bonding. In addition, choosing the use of aromatic hydrophobic groups might leverage π–π interactions, which may further strengthen the stacking of the cyclic peptides. We introduced a range of hydrophobic moieties (HMs: acetic acid (CH_3_), phenylacetic acid (Phe), cyclohexaneacetic acid (Cyclohex), octanoic acid (Hex), 1-naphthaleneacetic acid (Nap), 1-pyreneacetic acid (Pyr) and 1-adamantaneacetic acid (Ada), see Scheme 1) onto the cyclic peptides, and studied their influence of these hydrophobic groups on the self-assembly of CPPCs. Phe, Cyclohex and Hex, having the same number of carbon atoms, are expected to exhibit similar hydrophobicity, while with Phe providing the additional π–π stacking interactions of Phe is anticipated to further stabilize peptide assembly. Phe, Nap, and Pyr are also compared, to assess whether the increasing size of aromatic groups improves the self-assembly of CPPCs. A hydrophilic polymer, poly(ethylene glycol) (PEG), was used as a polymer graft to improve water solubility and prevent lateral aggregation.

## Results and Discussion

An asymmetric cyclic peptide, functionalized with both an amine group and an azide group, was designed to enable orthogonal coupling via strain-promoted alkyne–azide cycloaddition (SPAAC) and amidation, facilitating the conjugation of both a polymer and a hydrophobic moiety. To this end, a linear peptide, with the sequence H_2_N-l-Lys(Boc)-d-Leu-l-Trp(Boc)-d-Leu-l-Lys(N_3_)-d-Leu-l-Trp(Boc)-d-Leu-OH, was first synthesized by solid-phase peptide synthesis. The cyclization was achieved by dissolving the linear peptide in DMF and adding the coupling agent 4-(4,6-dimethoxy-1,3,5-triazin-2-yl)-4-methylmorpholinium chloride, tetrafluoroborate salt (DMTMM·BF_4_). The Boc-protecting groups were then cleaved from the cyclic peptides to yield the final cyclic peptide, as confirmed by ^1^H NMR spectroscopy and ESI-MS (Figure S1, S2 and Table S1).

**Figure 1:**
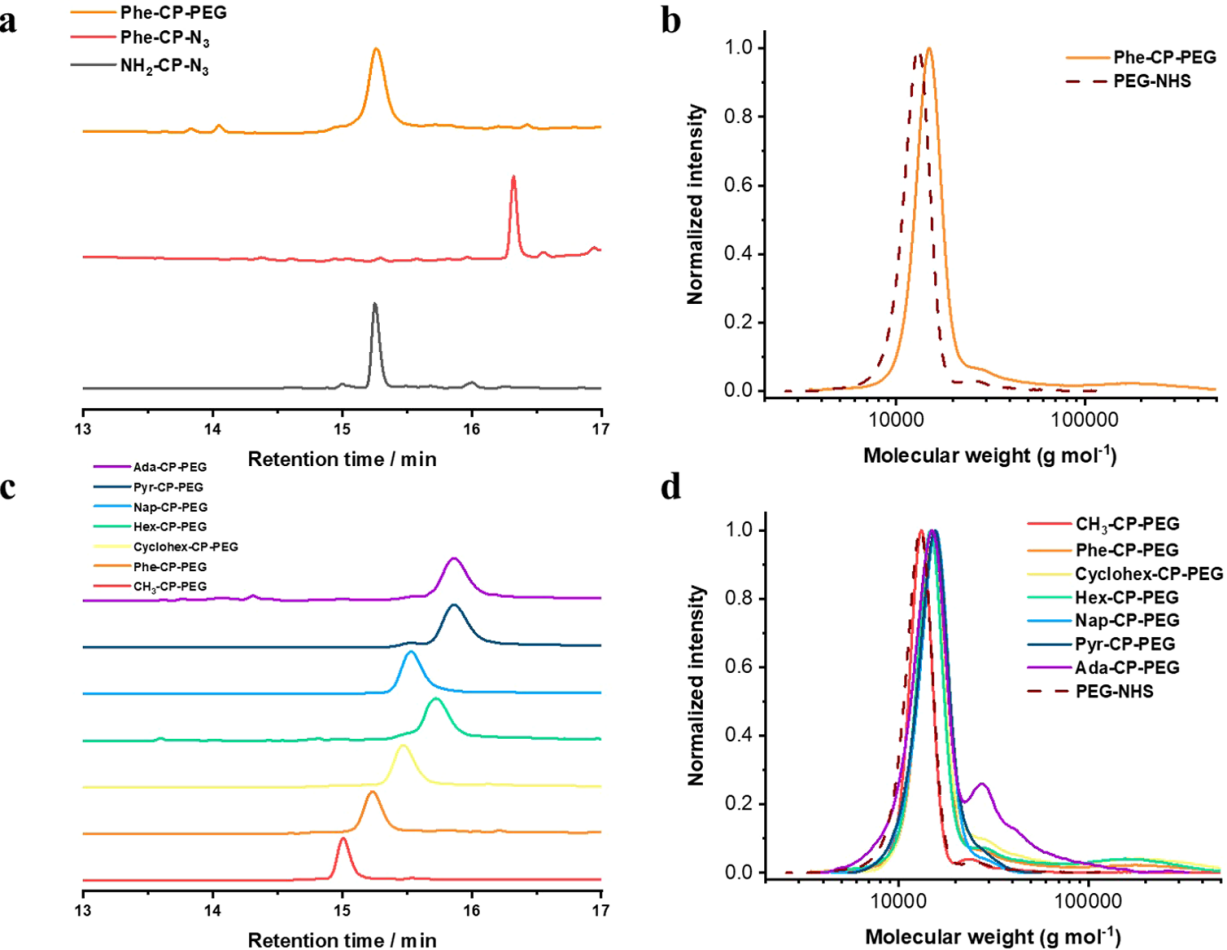
(a) HPLC spectra for free cyclic peptides, Phe-modified cyclic peptides, and Phe-CP-PEG conjugates; (b) GPC traces of free PEG and Phe-CP-PEG conjugates; (c) HPLC spectra for different HM-CP-PEG conjugates; (d) Normalized GPC traces of CP-PEG conjugates with different hydrophobic moieties. *M*_n_ and *Đ* values calculated from PMMA standards using DMF + 5 mM NH_4_BF_4_ as the eluent.

The synthesis of hydrophobic moiety-cyclic peptide–polymer conjugates was achieved by first reacting a hydrophobic moiety with the cyclic peptide via amidation reaction, followed by attaching a PEG chain to the hydrophobic moiety-cyclic peptide via alkyne–azide cycloaddition, resulting HM-CP-PEG (Scheme S2). Specifically, the attachment of hydrophobic moieties involved an amidation reaction between the carboxyl acid group of the hydrophobic molecules and the amine group on the cyclic peptide, in the presence of HATU coupling agent. These functionalized cyclic peptides were confirmed by ESI-MS and HPLC (Table S1 and Figure 1). A PEG chain (*M*_n_ = 5000 g mol^–1^) was first functionalized with a strained alkyne group, (1R,8S,9s)-bicyclo[6.1.0]non-4-yn-9-ylmethyl N-succinimidyl carbonate (BCN-NHS), followed by being conjugated to the azide-bearing CPs. Unreacted polymers were removed by fractional precipitation using a mixed solvent of DCM/diethyl ether.

HPLC and GPC were used to characterize the HM-CP-PEG conjugates. HPLC, equipped with a UV detector set to a wavelength of 280 nm, was used to detect the UV absorbance of tryptophan groups on the cyclic peptide cores. Taking Phe-CP-PEG as an example, as shown in Figure 1a, a new peak appeared at 16.4 min, corresponding to Phe-CP-N_3_. No unreacted cyclic peptides were detected at 15.2 min, indicating the complete functionalization of the cyclic peptides. After conjugation with PEG, a new peak was observed at 15.3 min, with no trace of unreacted Phe-CP-N_3_, confirming the purify of the Phe-CP-PEG conjugates. As indicated by GPC (Figure 1b), the conjugation of PEG-BCN (*M*_n_ = 12400 g mol^–1^ and *D* = 1.09) to the peptide led to a new peak corresponding to a *M*_n_ of 15800 g mol^–1^, further confirming the success of the coupling reaction, with no unreacted polymer left. Interestingly, a significant shoulder appeared at a higher molecular weight, likely due to the aggregation of the conjugates (Figure 1d).

Given the varying hydrophobicities of the synthesized HM-CP-PEG conjugates, HPLC is an ideal tool to differentiate the compounds. The Ada-CP-PEG and Pyr-CP-PEG conjugates exhibited long elution times, suggesting similar hydrophobicity. They both had the longest elution times among all conjugates, indicating higher hydrophobicity. The retention times for Phe-CP-PEG, Nap-CP-PEG, and Pyr-CP-PEG were 15.2, 15.5, and 15.9 min, respectively, suggesting that an increasing number of aromatic rings correlated with greater hydrophobicity. Interestingly, Phe-CP-PEG showed greater hydrophilicity than both Cyclohex-CP-PEG and Hex-CP-PEG. Lastly, CH_3_–CP-PEG had the lowest hydrophobicity, due to the presence of only a single methyl group (Figure 1c).

The assembly of HM-CP-PEG conjugates in water was investigated using a combination of small angle neutron scattering (SANS), transmission electron microscopy (TEM), and static light scattering (SLS) techniques.

**Figure 2:**
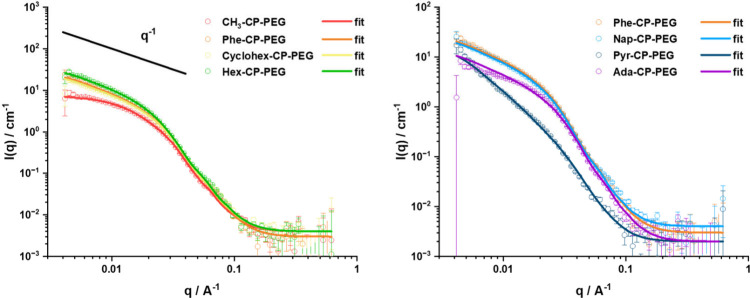
SANS scattering data for different HM-CP-PEGs and all fitting to a cylinder model (the slope of q^–1^ was showed for the comparison to the scattering intensity at low scattering angles).

First, the morphology and size of self-assembling HM-CP-PEGs in water were characterized by SANS (Figure 2 and S5). As reported previously,^20,25^ a q^–1^ dependency of the scattering intensity at low scattering angles is characteristic of a cylindrical structure, which was observed in all samples, confirming assembly into nanotubes. In addition, the q^–1^ dependency at lower q values suggests longer structures, allowing for the determination of nanotube lengths formed by each conjugate within the Guinier range.

For example, Hex-CP-PEG formed nanotubes longer than that of CH_3_–CP-PEG, as indicated by an intensity value of 28 cm^–1^, remarkably higher than that of CH_3_–CP-PEG (7 cm^–1^). Apart from CH_3_–CP-PEG, the scattering of all conjugates increased with a q^–1^ dependency at the lowest visible angle. Although there was no extended q^–1^ dependency observed for Pyr-CP-PEG at low q range, this behavior indicates the formation of long cylinders.^25^ However, we could not determine the exact length of these assemblies as they were outside of the SANS range (100 nm).

Using SasView software, the best-fit model for all systems was the core–shell cylinder model, confirming that these self-assembling conjugates formed cylindrical nanotubes in water. The fitted scattering length density (SLD) value of these conjugates was close to the calculated SLD value of solvent, indicating that the PEG chains were highly solvated in solution (Table S3). CH_3_–CP-PEG, showing the weakest hydrophobicity, was used as a control and was observed to form the shortest nanotubes in water, with a length of 36 nm (Chi^2^ 4.0), much shorter than the other systems, whose lengths all exceeded 100 nm—beyond the observable range in SANS analysis. As expected, the introduction of a hydrophobic group on the periphery of cyclic peptides strengthened the stability of these conjugates and further promoted their self-assembly.

**Figure 3:**
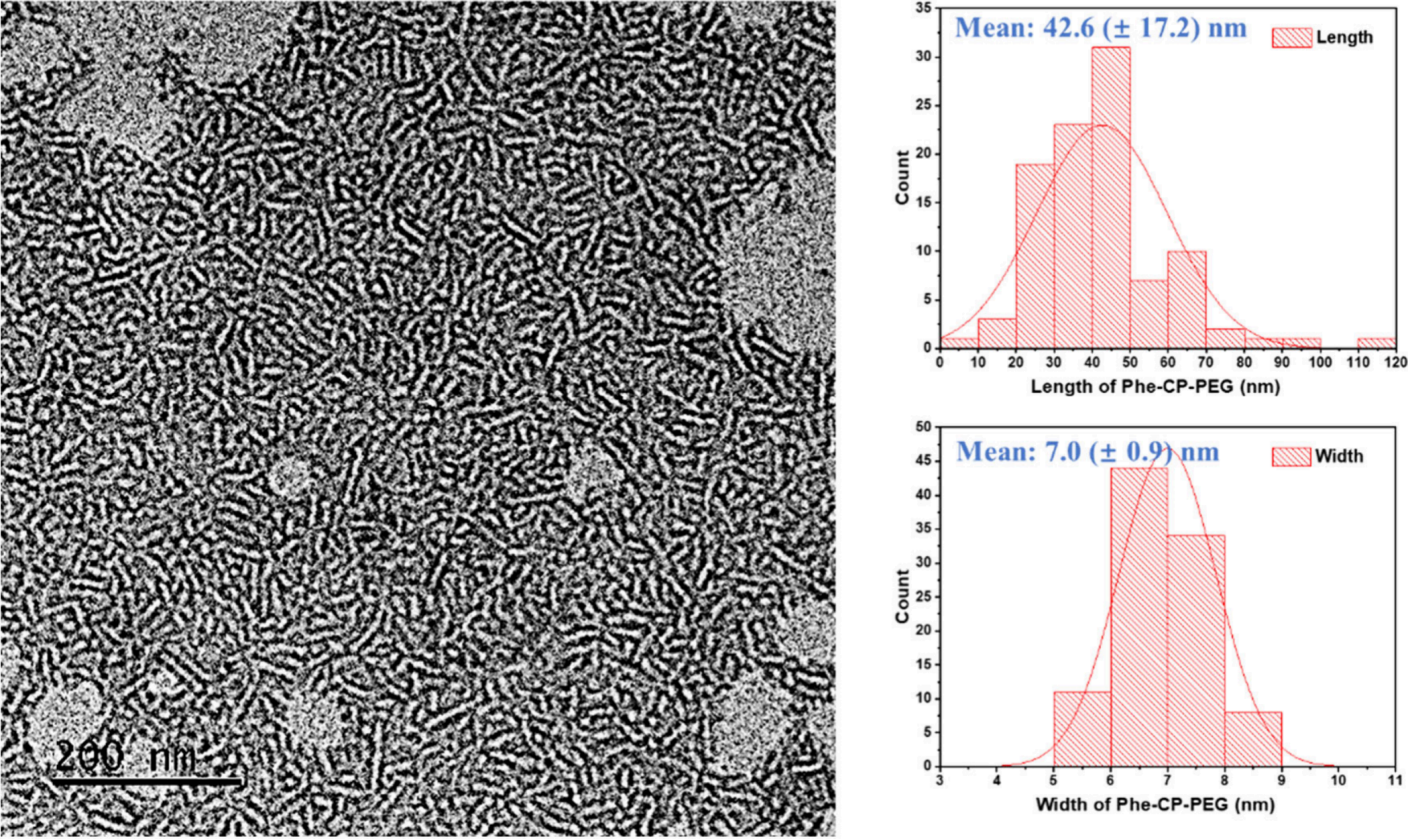
TEM data of the Phe-CP-PEG nanotubes.

TEM was used as a supplementary technique to confirm the morphology of the self-assemblies. All samples were stained by using a 0.2% uranyl acetate solution, which enabled the observation of 1D structures. Using ImageJ software, 100 individual tubular structures were randomly counted from a TEM image of each conjugate, and the distributions of the nanotube length and width were plotted (Figures 3 and S6).

For instance, Phe-CP-PEG exhibited an average length of 42.6 ± 17.2 nm, which is much smaller than that suggested by SANS, presumably due to the drying effect on TEM grids, which leads to the breakdown of the assemblies. However, the measured widths (e.g., 7.0 ± 0.9 nm for Phe-CP-PEG) are consistent with the formation of single nanotubes.

Considering the shortcomings of both SANS and TEM, we used static light scattering (SLS) to determine the precise length of the HM-CP-PEG nanotubes *in situ* in water. The SLS measurement can reflect the intensity of the scattered light (usually a visible laser source) as a function of scattering angle θ, by a sample in solution.^26^ For large aggregates with scattering angle dependency, SLS experiments are conducted with a range of sample concentrations in aqueous solution, which allows the construction of a Zimm plot.^27^ This plot enables the further extrapolation of the “zero-angle” intensity of a sample, providing the molecular weight of aggregates (*M*_a_) in solution. Given the known molecular weight of each cyclic peptide–polymer conjugate unimer, the aggregation number (*N*_agg_) of these assembling nanotubes could be calculated by dividing the observed *M*_a_ by the molecular weight of its corresponding unimer.^17^

Each conjugate was analyzed across a concentration gradient ranging from 1 to 5 mg mL^–1^ (Figure S7), and the results showed no concentration dependency.^28^ However, we could obtain the *M*_a_ of each conjugate under different concentration conditions (Table S4). According to the Zimm plot method, we further study the evolution of 1/*M*_a_ of HM-CP-PEGs as a function of concentration (Figure S8). In this case, we could extrapolate the *M*_a_ at “zero-angle” for each conjugate. Pyr-CP-PEG formed the longest assemblies in water, with a *M*_a_ of 3.1 × 10^7^ g mol^–1^ and an *N*_agg_ of 4811, while, as expected, CH_3_–CP-PEG formed the shortest assemblies, with a *M*_a_ of 1.4 × 10^6^ g mol^–1^ and an *N*_agg_ of 234. Considering the intersubunit distance of the cyclic peptides is 4.7 Å,^10^ the theoretical aggregation length of each conjugate could also be calculated (Table S5).

**Figure 4:**
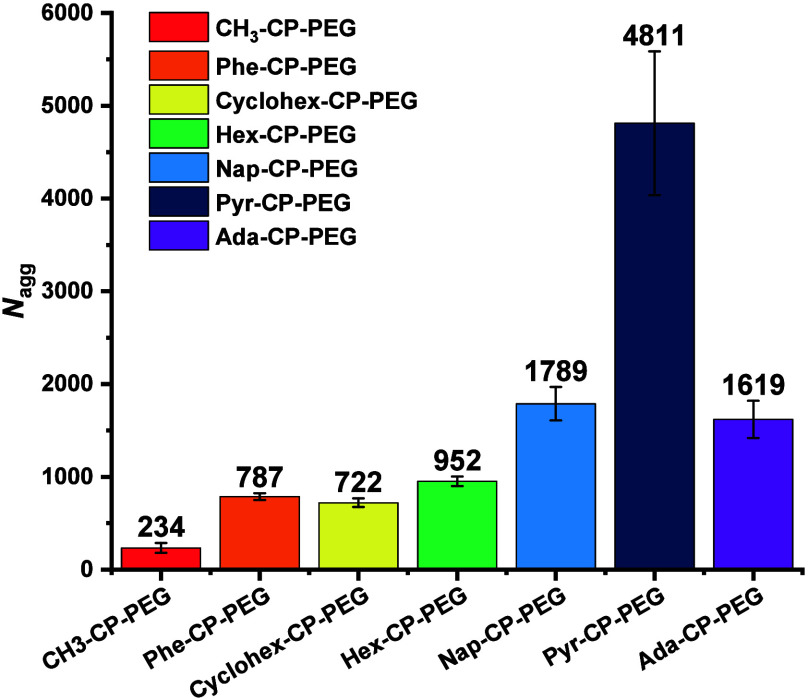
Summary of the aggregation number of each conjugate obtained by SLS.

As summarized in Figure 4, the self-assembly of HM-CP-PEG conjugates mainly depended on hydrogen bonding interactions associated with the cyclic peptide cores. Remarkably, the introduction of hydrophobic moieties enhanced their self-assembly behavior through additional hydrophobic interactions at the periphery.

In general, the more hydrophobic the conjugates are, the longer the assemblies they can aggregate into. The *N*_agg_ values for CH_3_–CP-PEG, Cyclohex-CP-PEG, Hex-CP-PEG and Ada-CP-PEG were 234, 722, 952, and 1619, respectively, corresponding to lengths of 110, 339, 447, and 761 nm, respectively (considering the spacing between two CPs is 4.7 Å^10^), which was consistent with the expected trend. Ada-CP-PEG showed a similar hydrophobicity with Pyr-CP-PEG, whereas its *N*_agg_ was far smaller than that of Pyr-CP-PEG, suggesting that π–π interactions from Pyr groups benefitted its self-assembly in water. Moreover, Phe-CP-PEG, despite having weaker hydrophobicity, formed longer assemblies than Cyclohex-CP-PEG, further highlighting the role of the π–π stacking, as seen in the comparison between Pyr-CP-PEG and Ada-CP-PEG. In water, aromatic molecules tend to stack due to the hydrophobic effect, because the solvation energy of aromatic surface is higher than that of bulk water, thus reducing the total surface exposed to solvent.^29^ A dramatic increasing trend of *N*_agg_ among Phe-CP-PEG, Nap-CP-PEG, and Pyr-CP-PEG was consistent with their increasing hydrophobicity trend. Besides, the existing π–π interactions may further enhance the stability of these supramolecular nanotubes in aqueous media.

## Conclusions

In conclusion, we successfully synthesized a series of cyclic peptide–polymer conjugates with various hydrophobic moieties. The introduction of hydrophobic groups onto the periphery of these conjugates significantly promoted their self-assembly in water by providing hydrophobic shielding for the cyclic peptides from water. Besides, π–π interactions between aromatic hydrophobic moieties further strengthened the assemblies. The synergistic effects of multiple driving forces, including hydrophobic interactions, π–π stacking, and multiple hydrogen bonding interactions, collectively strengthen the self-assembly of the cyclic peptide–polymer conjugates, enabling the formation of longer supramolecular nanotubes in water. This strategy allows for the tuning of polymeric nanotube length while maintaining their diameter and inherent properties. Furthermore, the incorporation of functional groups within the hydrophobic moieties—similar to those found in common anticancer drugs such as camptothecin and SN-38—could enrich the functionality of the supramolecular nanotubes for biological applications such as drug delivery and bioimaging.

## Supporting Information Available

Synthetic procedures and characterization data for all materials; small angle neutron scattering data and fit parameters; transition electron microscope images and analysis; static light scattering data (PDF)
