# Supplementary material for: Strengthening the Self-Assembly of Supramolecular Polymeric Nanotubes in Water via the Introduction of Hydrophobic Moieties
Source: ACS Macro Lett. 2025 Feb 21;14(3):292–8. doi: 10.1021/acsmacrolett.4c00759 (PMC11924312; doi:10.1021/acsmacrolett.4c00759)
Supplement: Supplementary file 1 — mz4c00759_si_001.pdf [file mz4c00759_si_001.pdf]

Supporting information

Strengthening the Self-assembly of Supramolecular Polymeric Nanotubes in Water *via* the Introduction of Hydrophobic Moieties

Zihe Cheng, [a] *Stephen C. L. Hall,* [b] *Qiao Song,* [c] *and Sébastien Perrier* [*a,d,e]

a. Department of Chemistry, University of Warwick, Coventry CV4 7AL, UK.

b. ISIS Neutron and Muon Source, Rutherford Appleton Laboratory, Didcot OX11 0QX

c. Shenzhen Grubbs Institute, Southern University of Science and Technology, Shenzhen 518055, China

d. Warwick Medical School, University of Warwick, Coventry CV4 7AL, UK.

e. Faculty of Pharmacy and Pharmaceutical Sciences, Monash University, Parkville, VIC 3052, Australia.

* Corresponding author: s.perrier@warwick.ac.uk

1. Materials and Characterization

Materials

2-(6-chloro-1-*H*-benzotriazole-1-yl)-1,1,3,3-tetramethylaminium hexafluorophosphate (HCTU) and Fmoc-protected amino acids were purchased from Iris Biotech. 1-[Bis(dimethylamino) methylene]-1*H*-1,2,3-triazolo[4,5-*b*] pyridinium 3-oxid hexafluorophosphate (HATU) was purchased from Alfa Aesar. *m*PEG5k-NH_2 was purchased from Rapp Polymere. All other chemicals and solvents were purchased from Sigma-Aldrich.

Characterization

Nuclear Magnetic Resonance Spectroscopy (NMR): ^{1}H NMR spectra were measured using a Bruker Avance III HD 400 MHz NMR spectrometer. Polymers were dissolved in deuterated water (D_2O). Peptides and conjugates were dissolved in deuterated trifluoroacetic acid (*d*-TFA) and (Methyl sulfoxide)-d6 (*d*-DMSO). The residual solvent peaks were used as internal references.

High-Performance Liquid Chromatography (HPLC): HPLC was measured using a Shimadzu Prominence HPLC, equipped with a Luna C18 100 Å column (250 mm × 4.6 mm). the mobile phase was acetonitrile/water. All samples were prepared in pure water with the addition of 0.04 vol% TFA. Detection was equipped to monitor UV absorption at 280 nm and 309 nm.

Gel Permeation Chromatography (GPC): GPC was measured using an Agilent Infinity II MDS instrument equipped with such various detectors as differential refractive index (DRI), ultraviolet (UV) and viscometry (VS). Two PolarGel H columns (300 mm × 7.5 mm) and a PolarGel 5 µm guard column were included in the system calibrated using poly(methyl methacrylate). The eluent of this system is DMF with 5 mmol NH_4BF_4 additive. NH_4BF_4 is an

electrolyte which reduces the electrostatic interactions between polymer molecules and the column packing or between different polymeric conjugates which could cause aggregation, ensuring a more accurate size-based separation. It also reduces peak broadening or tailing, allowing for better resolution. All samples were prepared by dissolving in DMF and then filtered by 0.22 μm pore size PTFE membranes before measurement. Exported data were processed by the Agilent GPC/SEC software.

Electron Spray Ionization Mass Spectrometry (ESI-MS): ESI-MS was measured using an Agilent 6130B instrument with positive and negative ionization modes. All samples were prepared in methanol and then filtered by 0.2 μm pore size PTFE membranes before measurement.

Static Light Scattering (SLS): SLS was measured using an ALV/CGS-3 Compact Gonimeter instrument equipped with a wavelength of 632 nm polarized laser at 25 °C. All conjugate samples were prepared in water with a range of concentration gradients: 1.0-5.0 mg/mL. Before measurement, all sample solutions were filtered by 0.45 μm pore size Nylon membranes.

Transmission electron microscope (TEM): 10 μL aqueous solution of conjugates was drop-casted on the TEM grid with carbon-coat. After 3 min, the excess solution was absorbed with filter paper. The sample grid was left for 15 min and then stained using a 0.2% uranyl acetate solution (10 μL) for 30 s. Bright field of TEM micrographs were obtained using a 200kV operated JEOL 2100Plus microscope with a Gatan OneView IS camera.

Small Angle Neutron Scattering (SANS):

SANS was achieved on the LARMOR small angle diffractometer at the ISIS Pulsed Neutron Source (STFC Rutherford Appleton Laboratory, Didcot, UK). Before measurement, all samples were dissolved in D_2O with 5% DMSO and placed in a 2mm quartz cuvette. The scattering cross-section was measured over a Q-range of 0.004 - 0.5 $Å^{-1}$ where Q is defined as:

$$Q = \frac{4\pi sin\frac{\theta}{2}}{\lambda}$$

Here, θ is the scattered angle, and λ is the incident neutron wavelength. A Q-range of 0.004 - 0.5 $Å^{-1}$ was achieved utilizing an incident wavelength range of 0.9 - 13.3 Å. The detector is located 4.1 m from the sample and is 664 mm wide × 664 mm high with the beam in the centre of the detector. The beam size is 6 mm wide and 8 mm high. Each raw scattering data set was corrected for the detector efficiencies, sample transmission and background scattering and converted to scattering cross-section data ($\partial\Sigma/\partial\Omega$ vs. Q) using the instrument-specific software. These data were placed on an absolute scale (cm^{-1}) using the scattering from a standard sample (a solid blend of hydrogenous and perdeuterated polystyrene) in accordance with established procedures.

2. Synthesis

Peptide synthesis

The synthetic method for this cyclic peptide has been reported by the Perrier group. [1]

Linear peptide synthesis

The linear peptide, with the sequence of H_2N-L-Lys(Boc)-D-Leu-L-Trp(Boc)-D-Leu-L-Lys(N_3)-D-Leu-L-Trp(Boc)-D-Leu-OH was synthesized by solid phase peptide synthesis on a Prelude Automated Peptide Synthesizer. 2-chlorotrityl chloride resin worked as a solid support. The first Fmoc-protected amino acid was attached to the resin by using DIPEA (4 eq) in DCM, followed by capping of unreacted resin sites using a mixture solution of MeOH:DIPEA:DCM (7:1:2, v/v/v). Deprotection of the Fmoc group of amino acids was achieved using 20% piperidine in DMF. Subsequent amino acids were coupled using Fmoc-amino acids (5 eq), HCTU (5 eq) and NMM (10 eq) in DMF. The linear peptide was cleaved from the resin by using 20% HFIP in DCM solution and finally dried in a vacuum oven to obtain a white solid. Yield: 498.9 mg.

Cyclization

The cyclization of linear peptide was done by using a coupling agent, DMTMM·BF_4. Specifically, linear peptide (1 eq, 498.9 mg, 0.350 mmol) and DMTMM·BF_4 (1.2 eq, 137.7 mg, 0.420 mmol) was dissolved in 100 mL and 10 mL DMF respectively. Then solutions were mixed into a 250 mL round bottle flask equipped with a stirring bar and stirred continuously for 96 h at room temperature. After that, the solution was concentrated to around 5 mL by a rotary evaporation (50°C) and then washed three times by cold methanol/water = 1/1. The precipitate was dried in a vacuum oven to an off-white solid. Yield: 455.5 mg (91%).

Deprotection

The Boc deprotection was done by adding a mixture solution of TFA: TIPS: H_2O (1.8 mL: 0.1 mL: 0.1 mL) to protected peptides and stirring for 3 h at room temperature. The resulting solution was precipitated twice using cold diethyl ether. The obtained white solid was the deprotected cyclic peptide and then dried in a vacuum oven (40 °C, 3 hours). Yield: 396.9 mg (87%).

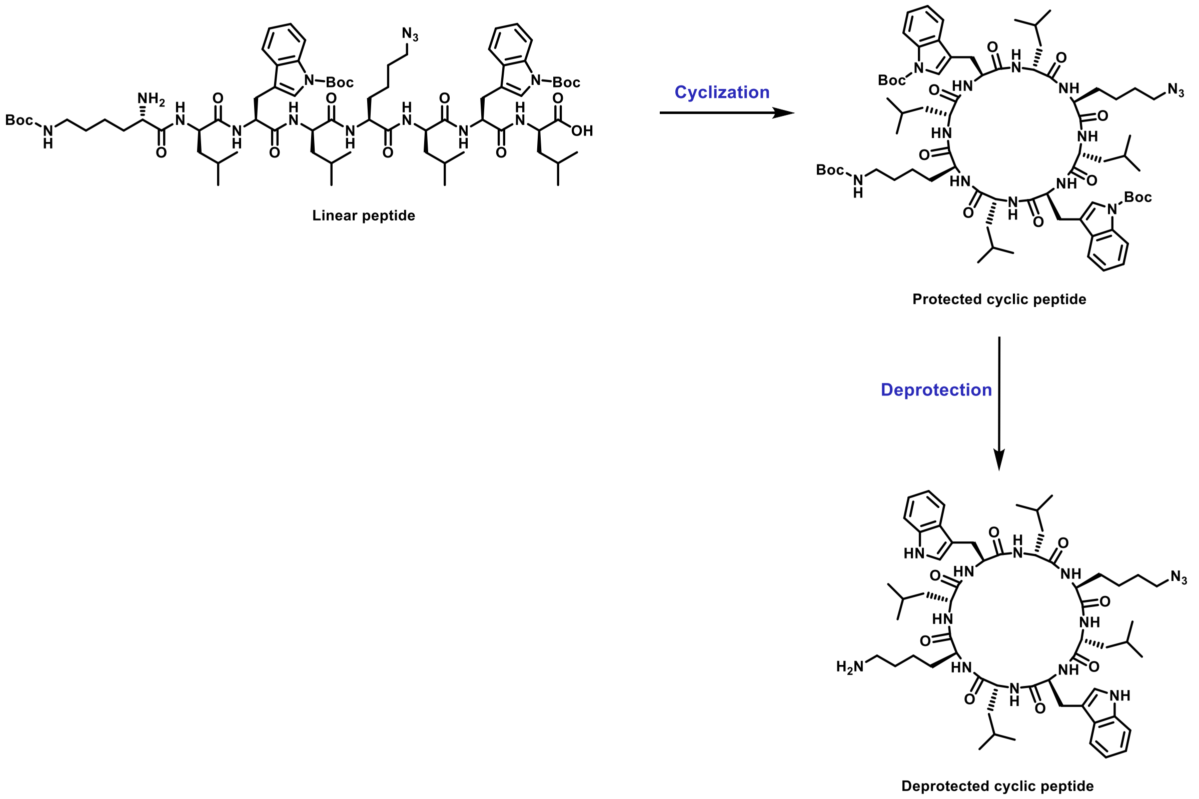

Scheme S1: Synthetic pathway of cyclic peptides.

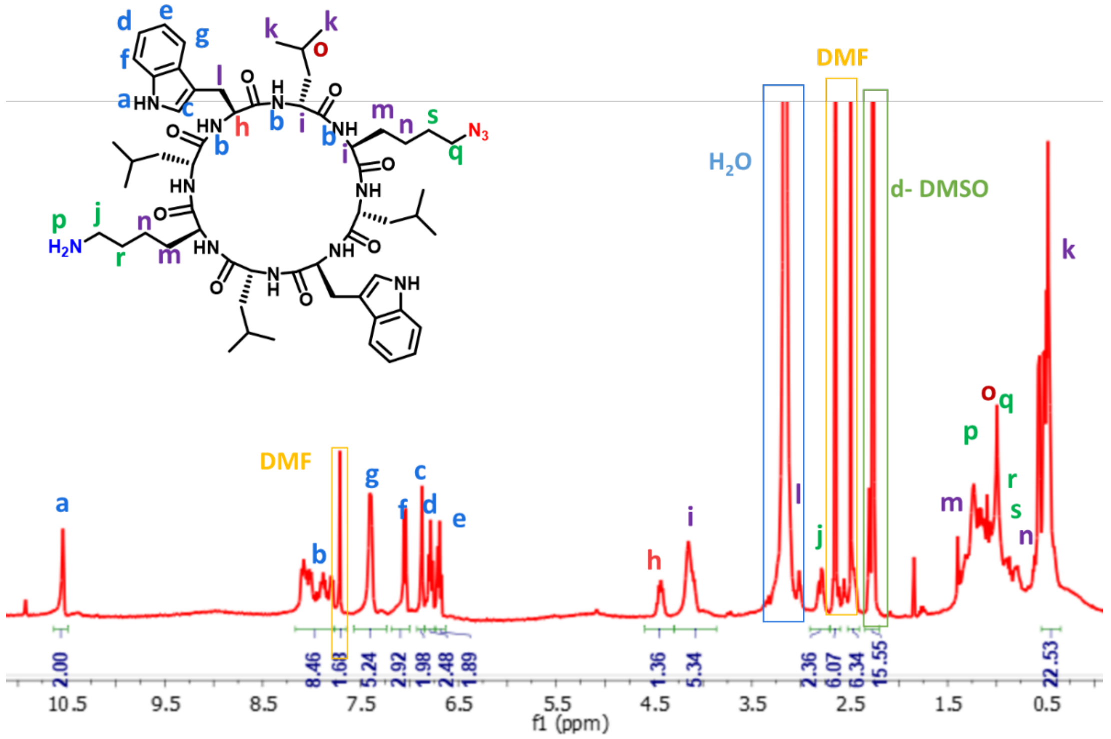

Figure S1: ^{1}H NMR spectrum of cyclic peptides (400 MHz, *d*-DMSO).

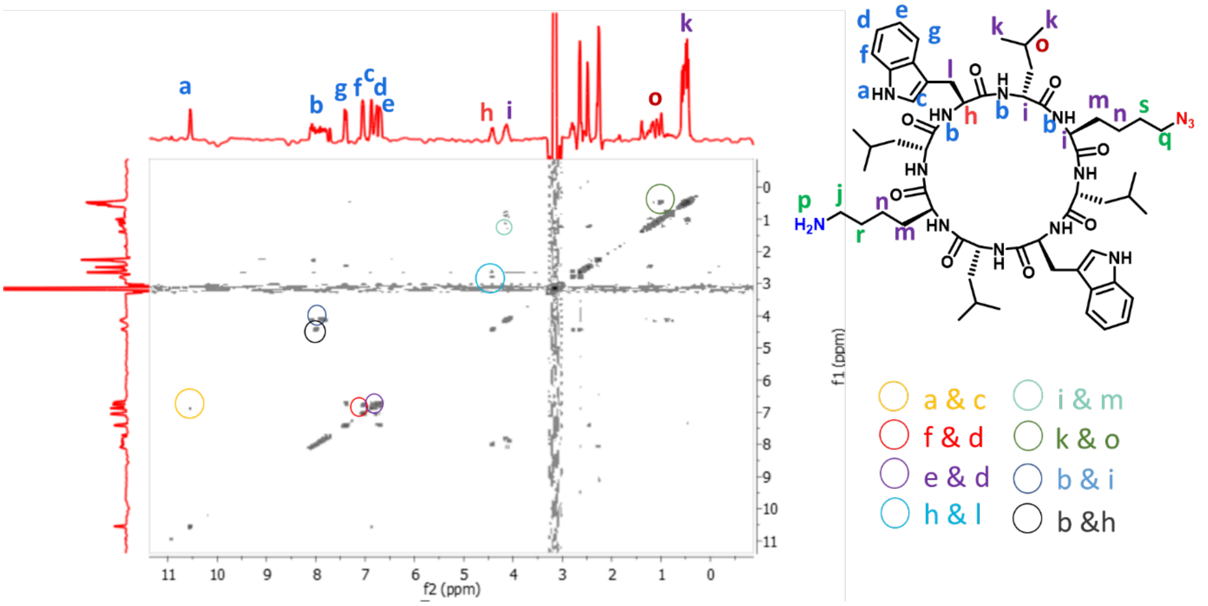

Figure S2: ^{1}H-^{1}H COSY NMR spectrum of cyclic peptides (400 MHz, *d*-DMSO).

Synthesis of functionalized cyclic peptide polymer conjugates

Hydrophobic conjugates were synthesized by the same strategy. Here we took the Phe-CP-PEG conjugate as an example.

Synthesis of mPEG-BCN

249.6 mg of mPEG-NH_2 (M_n=5000 g mol^{-1}, 1 eq, 50 µmol) and 21.9 mg of BCN-NHS (1.5 eq, 75 µmol) were dissolved in 0.8 mL and 0.2 mL DMF respectively. Solutions were transferred into one vial after they were fully dissolved. Then, 303.4 µL of NMM stock solution (50 mg/mL, 3 eq, 150 µmol) were added. The reaction was left at room temperature for 1 day. The obtained mixture was added dropwise into cold diethyl ether and then redissolved in DMF and washed twice by cold ether. DMF and excess reactants were removed with supernatant *via* centrifugation (7800 rpm, 5 min). The white solid precipitate was deposited at the bottom of tube and dried under N_2. Yield: 214.4 mg. ^{1}H NMR (400 MHz, D_2O, ppm) δ = 4.10 (m, 2H), 3.18 (m, 3H), 3.15 (m, 2H), 2.20-2.05 (m, 4H), 1.55 (m, 4H), 1.35 (m, 1H), 0.90 (m, 2H).

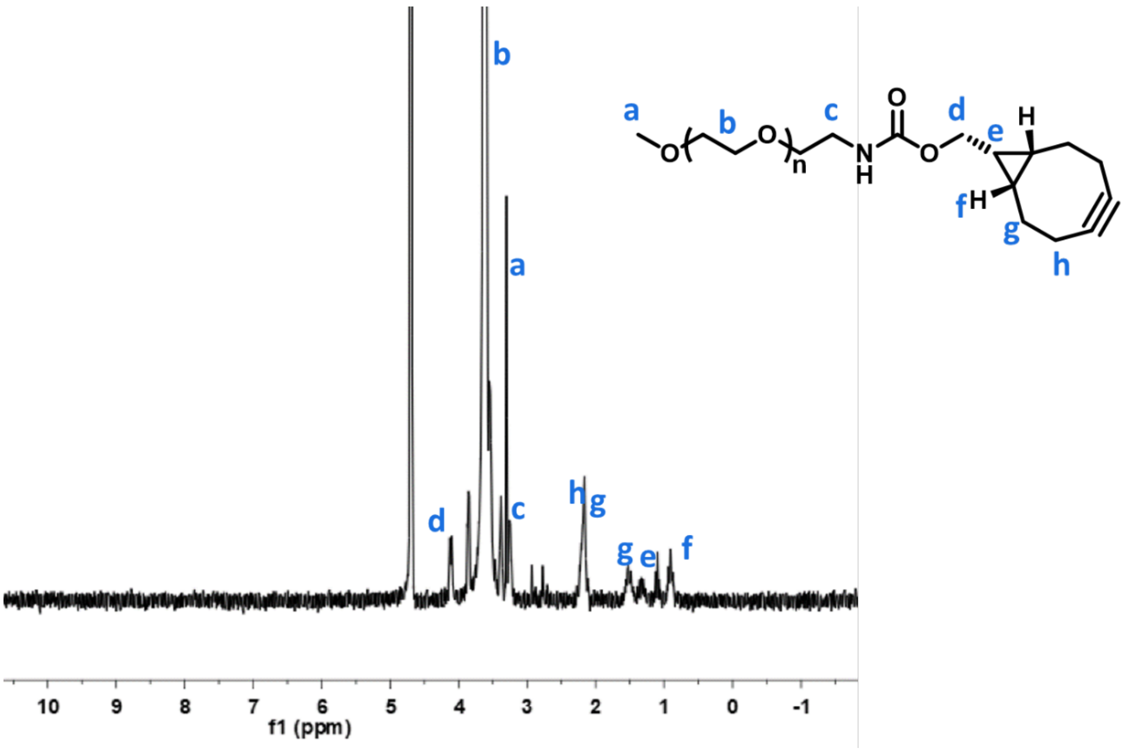

Figure S3: ^{1}H NMR spectrum of mPEG-BCN (400 MHz, D_2O).

Phe-CP-N₃

20.1 mg of CP_2 (1 eq, 18.06 µmol) and 4.6 mg of phenylacetic acid (2 eq, 36.12 µmol) were dissolved in 0.5 mL and 0.5 mL DMF respectively. Solutions were transferred into a 5 mL vial after they were entirely dissolved *via* sonication. Then, 124.5 µL of HATU stock solution (100 mg/mL, 2 eq, 36.12 µmol) and 99.4 µL of NMM stock solution (50 mg/mL, 3 eq, 54.18 µmol) were introduced. The mixture was reacted at room temperature for 72 hours. Due to the great solubility of phenylacetic acid in diethyl ether, functionalised cyclic peptide was washed twice using cold diethyl ether. The precipitate was collected and dried under N_2, then dried in a vacuum oven (40 °C, 1 hour).

Phe-CP-PEG

10.0 mg of Phe-CP-N_3 (1 eq, 8.16 µmol) and 63.7 mg of mPEG-BCN (1.5 eq, 12.24 µmol) were dissolved in 0.5 mL and 0.5 mL DMF respectively. Solutions were transferred into one vial and then left at room temperature for 2 days. The reaction mixture was added dropwise into cold diethyl ether in a centrifuge tube. The precipitate was collected and dried under N_2. Then, the solid mixture was redissolved in 1.5 mL DCM and 8.25 mL diethyl ether was added

proportionally. This step was repeated twice, and excess unreacted PEGs were removed with the supernatant. The precipitate was collected and dried under N_2.

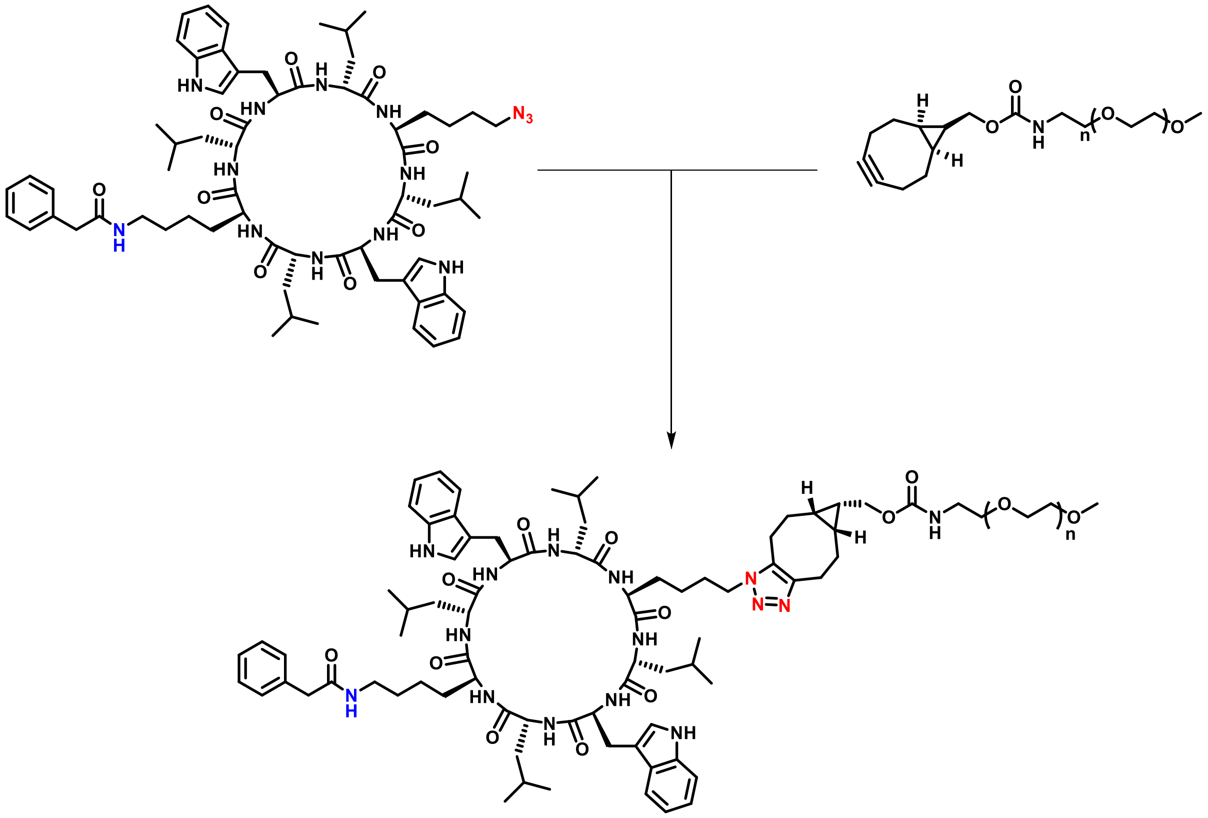

Scheme S2: Synthetic strategy of Phe-CP-PEG.

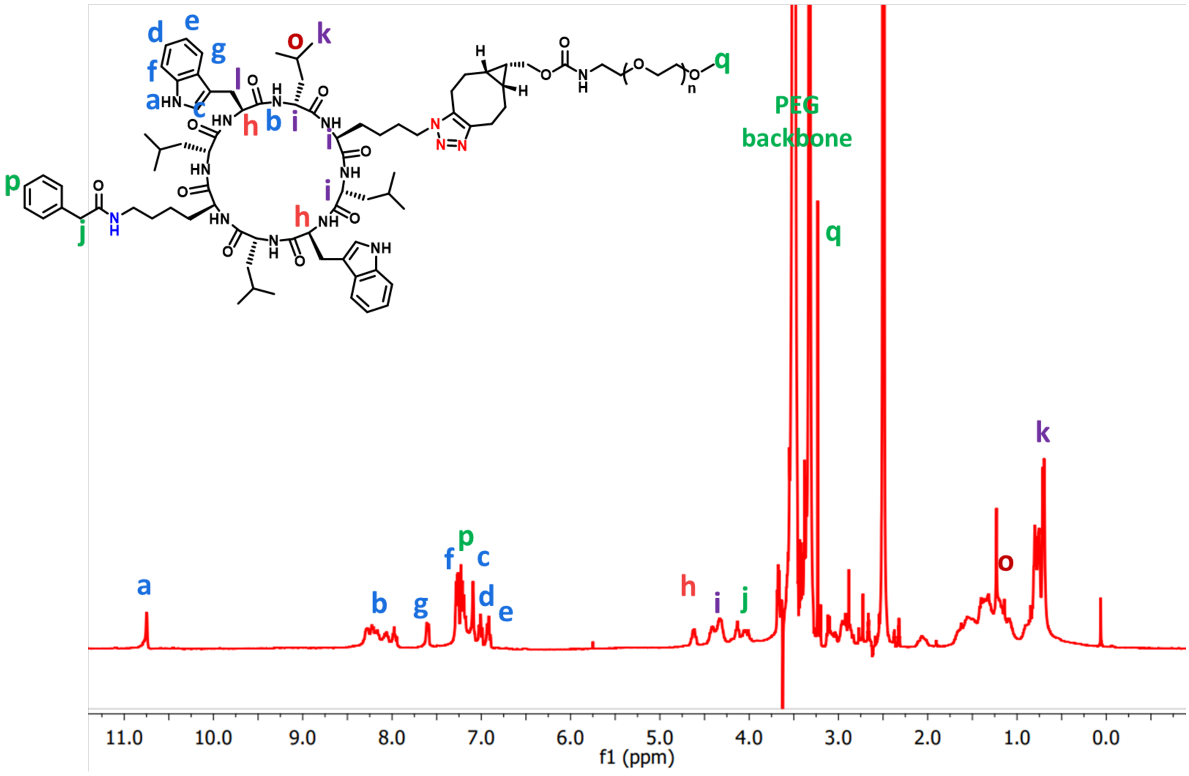

Figure S4: ^{1}H NMR spectrum of Phe-CP-PEG (400 MHz, *d*-DMSO).

Table S1. Summary of ESI-MS data for peptide at each step using an Agilent 6130B instrument with positive and negative ionization modes (800-2000 m/z).

Sample	ESI (g mol^{-1})		
	**Calculated**	**Found**	**Attribution**
Linear peptide	1448.8	1448.7	$[M+Na]^+$
	1424.8	1424.7	$[M-H]^-$
Boc-protecting cyclic peptide	1430.8	1430.7	$[M+Na]^+$
	1442.8	1442.7	$[M+Cl]^-$
Cyclic peptide	1130.4	1130.7	$[M+Na]^+$
	1106.4	1106.7	$[M-H]^-$
CH_3-CP-N_3	1171.5	1171.7	$[M+Na]^+$
	1183.5	1183.5	$[M+Cl]^-$
Phe-CP-N_3	1248.6	1248.7	$[M+Na]^+$
	1260.6	1260.6	$[M+Cl]^-$
Cyclohex-CP-N_3	1254.6	1254.7	$[M+Na]^+$
	1266.6	1266.7	$[M+Cl]^-$
Hex-CP-N_3	1256.6	1256.8	$[M+Na]^+$
	1268.6	1268.6	$[M+Cl]^-$
Nap-CP-N_3	1297.7	1297.7	$[M+Na]^+$
Pyr-CP-N_3	1371.7	1371.6	$[M+Na]^+$
Ada-CP-N_3	1305.6	1306.6	$[M+Na]^+$

Table S2. Summary of GPC data for synthetic HM-CP-PEG conjugates with different hydrophobic moieties.

**Sample**	**M_n (g mol^{-1})**	**Đ**
BCN-PEG	12400	1.09
CH_3-CP-PEG	13200	1.17
Phe-CP-PEG	15800	2.00
Cyclohex-CP-PEG	17600	2.52
Hex-CP-PEG	16300	2.17
Nap-CP-PEG	14700	1.06
Pyr-CP-PEG	14800	1.07
Ada-CP-PEG	15800	1.41

Small angle neutron scattering (SANS)

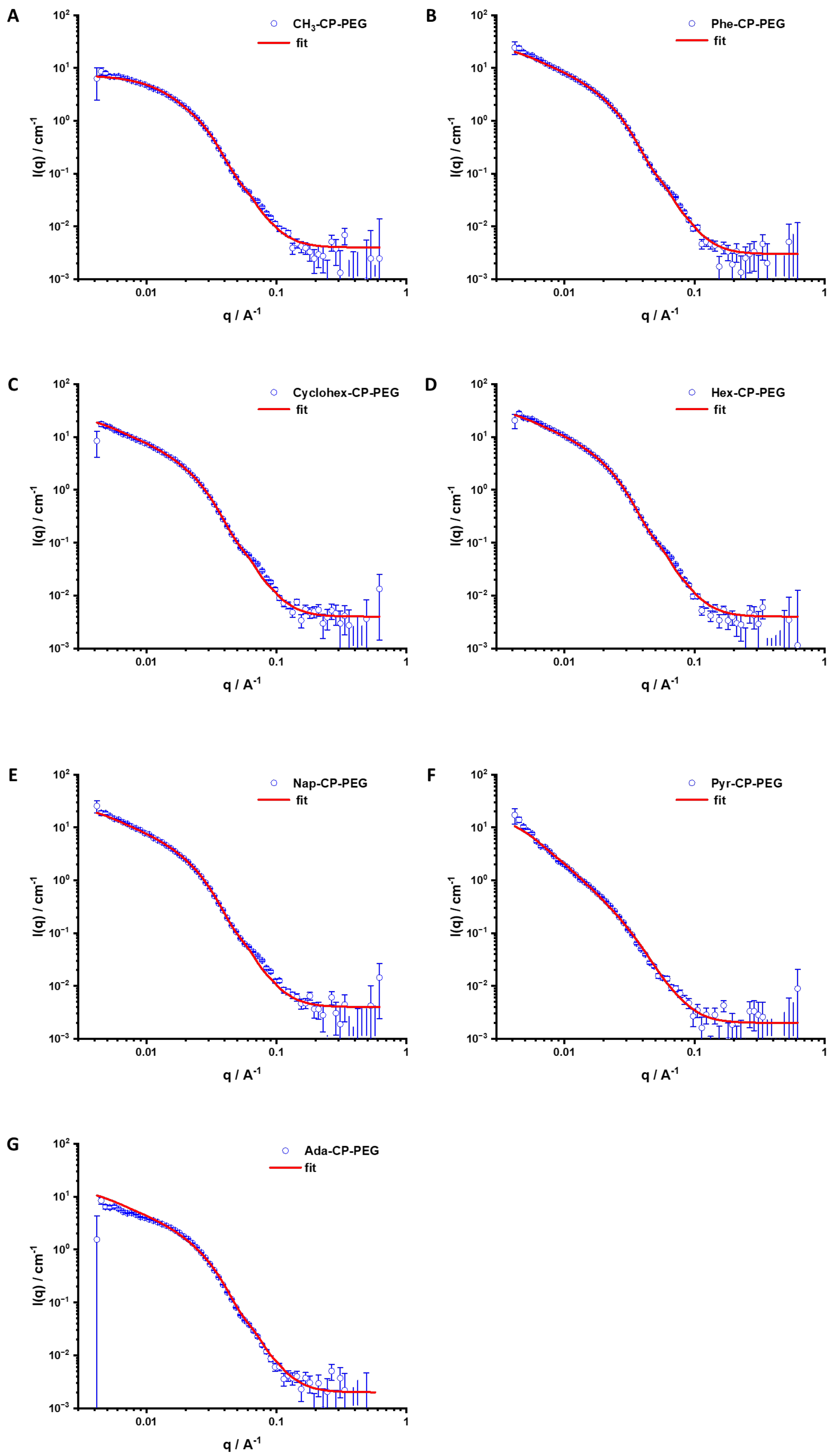

Figure S5. SANS data for each hydrophobic conjugate fitted to a cylinder model by Sasview software. A) CH_3-CP-PEG; B) Phe-CP-PEG; C) Cyclohex-CP-PEG; D) Hex-CP-PEG; E) Nap-CP-PEG; F) Pyr-CP-PEG; G) Ada-CP-PEG.

Table S3. SANS data for HM-CP-PEG conjugates with different hydrophobic moieties.

SLD_solvent – calculated using molecular structure and density.

Radius – set at 5Å as the radius of a free cyclic peptide.

CH_3-CP-PEG (Cylinder model)

Parameters	Fit	Value	units
scale	√	0.14649	
background		0.004	1/cm
sld	√	6.4644	$1e^{-6}$/Å^2
sld_solvent		6.1348	$1e^{-6}$/Å^2
radius		5	Å
length	√	361.68	Å
Reduced Chi^2		4.00	

Phe-CP-PEG (Cylinder model)

Parameters	Fit	Value	units
scale	√	0.0079091	
background		0.003	1/cm
sld	√	7.8111	$1e^{-6}$/Å^2
sld_solvent		6.1348	$1e^{-6}$/Å^2
radius		5	Å
length	√	1000	Å
Reduced Chi^2		3.12	

Cyclohex-CP-PEG (Cylinder model)

Parameters	Fit	Value	units
scale	√	0.035467	
background		0.004	1/cm
sld	√	6.9168	$1e^{-6}$/Å^2
sld_solvent		6.1348	$1e^{-6}$/Å^2
radius		5	Å
length	√	1000	Å
Reduced Chi^2		4.91	

Hex-CP-PEG (Cylinder model)

Parameters	Fit	Value	units
scale	√	3.1521	
background		0.004	1/cm
sld	√	6.2246	$1e^{-6}$/Å^2
sld_solvent		6.1348	$1e^{-6}$/Å^2
radius		5	Å
length	√	1000	Å
Reduced Chi^2		5.26	

Nap-CP-PEG (Cylinder model)

Parameters	Fit	Value	units
scale	√	0.060695	
background		0.004	1/cm
sld	√	6.7222	$1e^{-6}/Å^2$
sld_solvent		6.1348	$1e^{-6}/Å^2$
radius		5	Å
length	√	1000	Å
Reduced Chi^2		5.20	

Pyr-CP-PEG (Elliptical cylinder)

Parameters	Fit	Value	units
scale	√	0.019751	
background		0.002	1/cm
radius_minor	√	5.0745	Å
axis_ratio	√	7.5613	
length	√	1000	Å
sld	√	6.6149	$1e^{-6}/Å^2$
sld_solvent		6.1348	$1e^{-6}/Å^2$
Reduced Chi^2		4.23	

Ada-CP-PEG (Cylinder model)

Parameters	Fit	Value	units
scale	√	0.03973	
background		0.002	1/cm
sld	√	6.7627	$1e^{-6}/Å^2$
sld_solvent		6.1348	$1e^{-6}/Å^2$
radius		5.0	Å
length	√	1000	Å
Reduced Chi^2		19.78	

The individual fit was considered statistically reliable when the reduced Chi^2 (<30) was obtained.

Transmission electron microscope (TEM)

The length and width of different conjugates were measured by counting 100 units from each image. Nap-CP-PEG, Pyr-CP-PEG and Ada-CP-PEG images were hard to identify individual lengths due to high concentration. So, we count each width only.

Cyclohex-CP-PEG5K

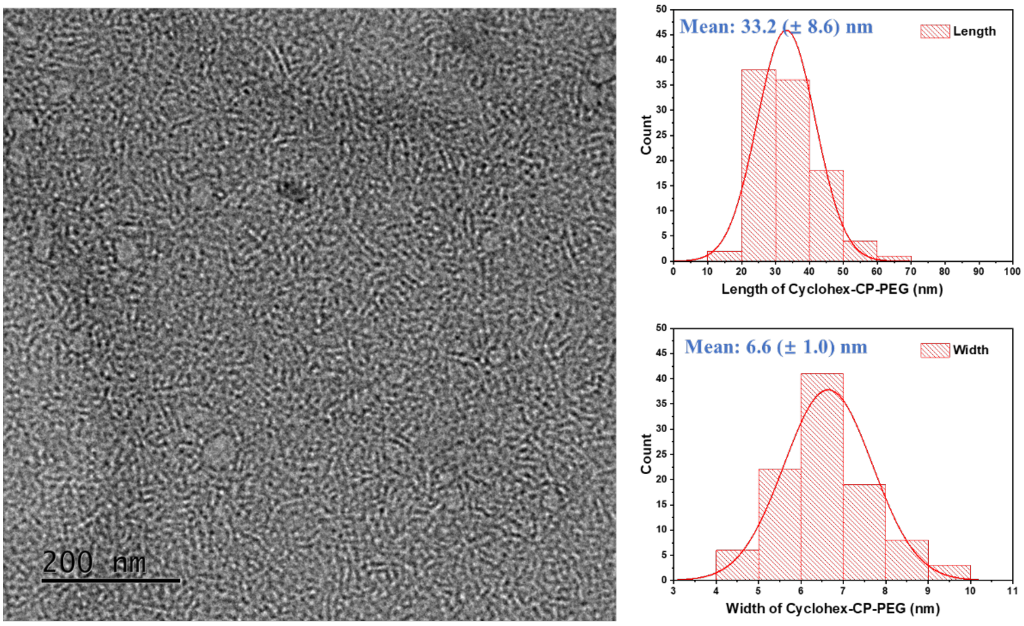

Hex-CP-PEG5K

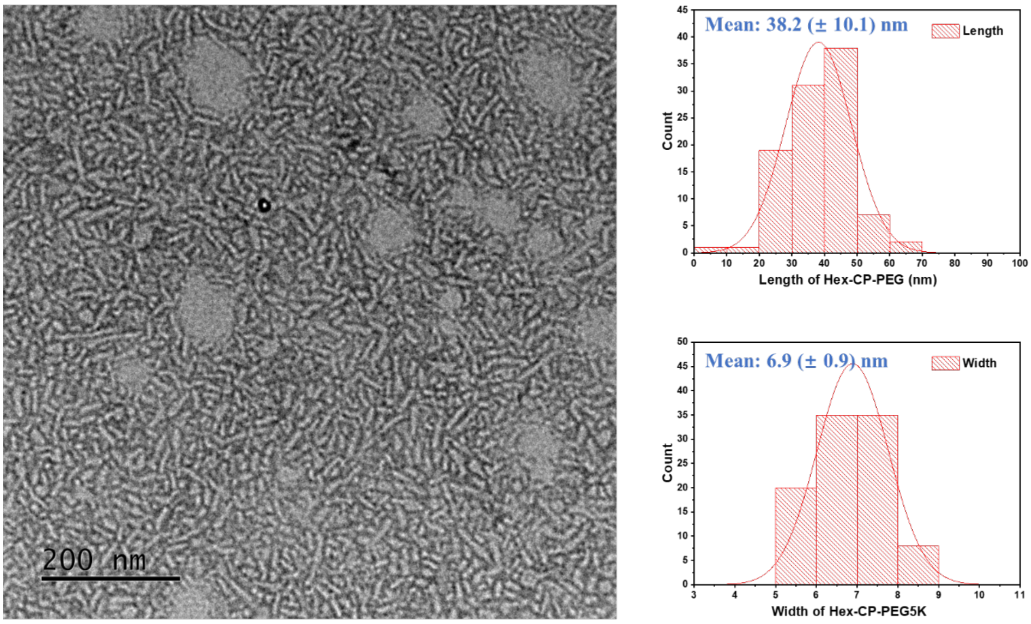

Nap-CP-PEG5K

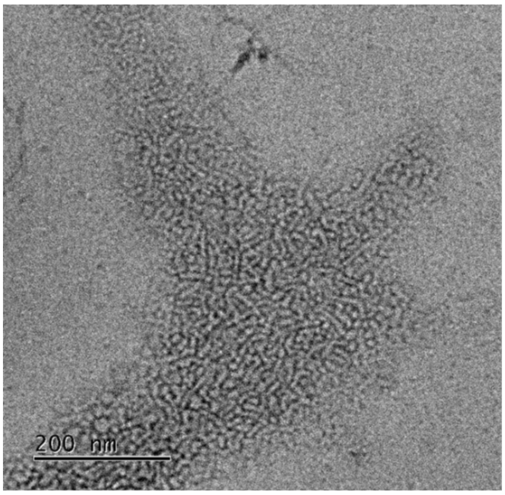

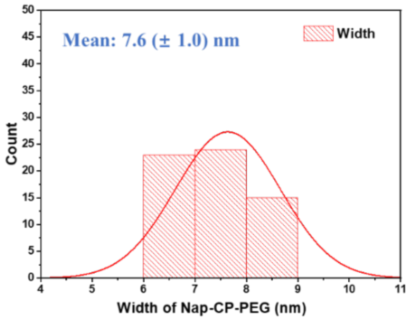

Pyr-CP-PEG5K

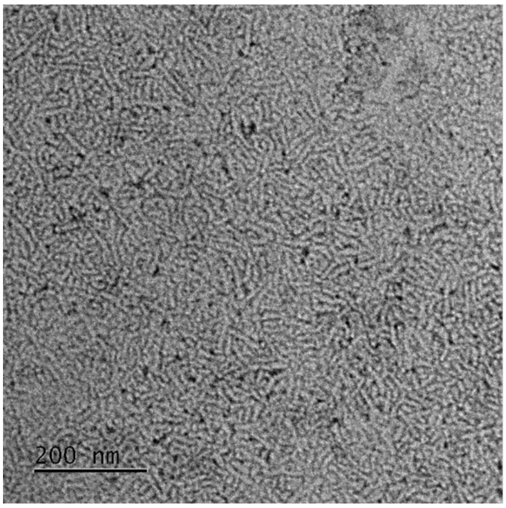

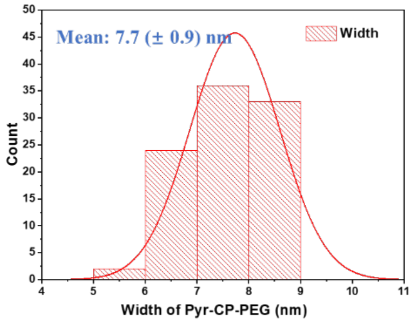

Ada-CP-PEG5K

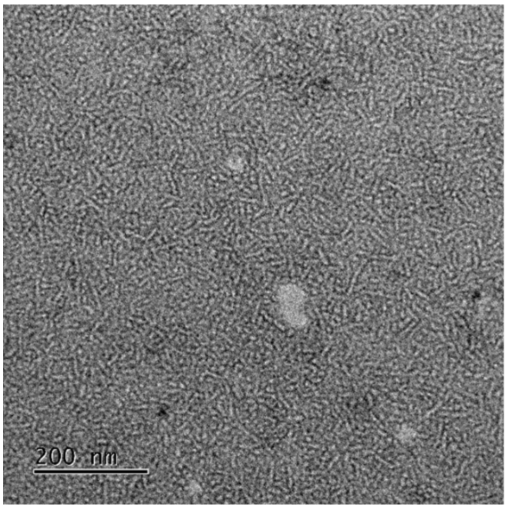

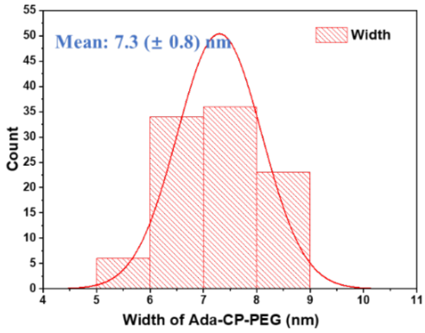

Figure S6. Summary of TEM data for different conjugates.

Static light scattering (SLS)

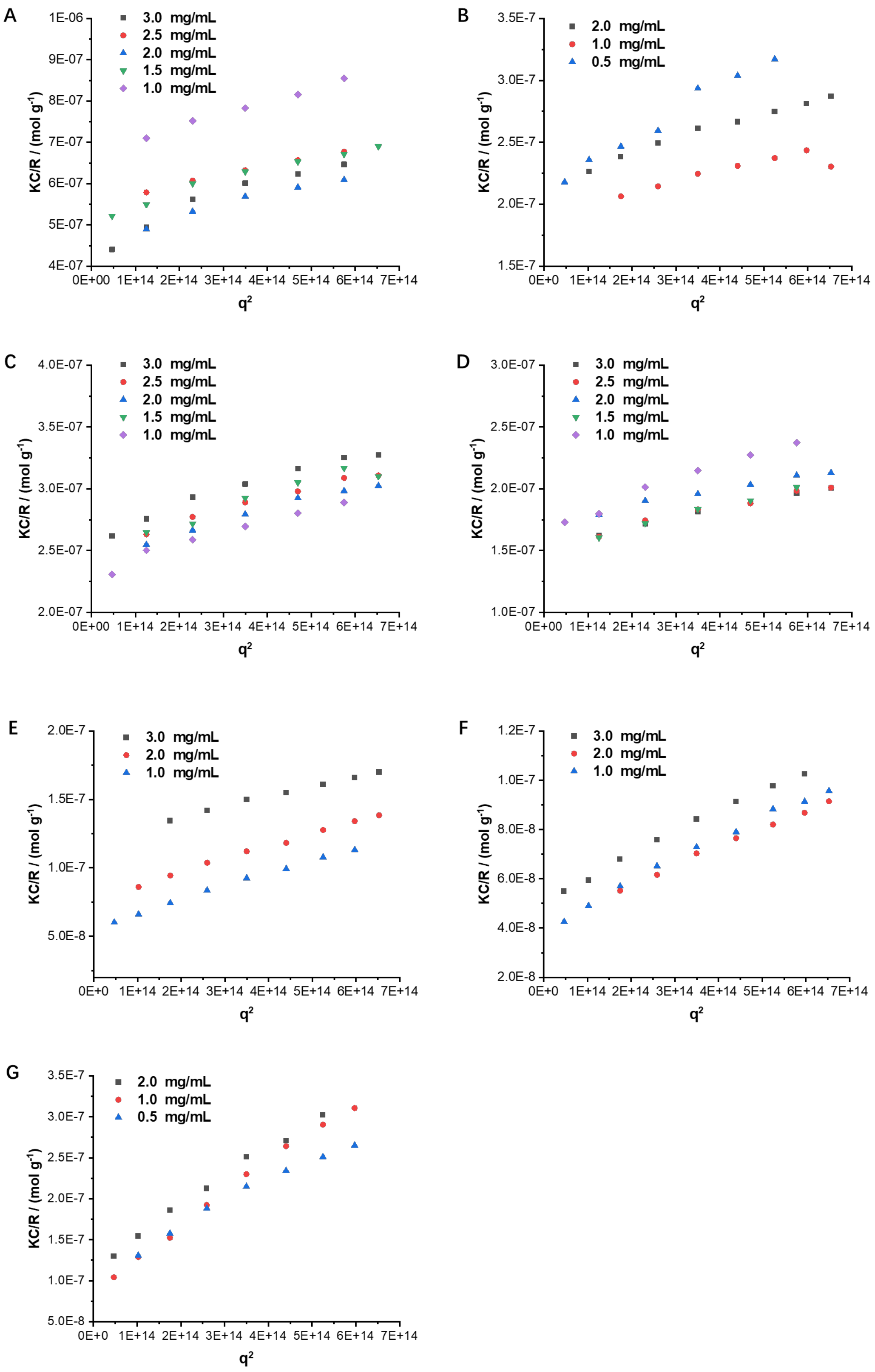

Figure S7. Evolution of KC/R of different HM-CP-PEGs in water as a function of q^2 measured by SLS. A) CH_3-CP-PEG; B) Phe-CP-PEG; C) Cyclohex-CP-PEG; D) Hex-CP-PEG; E) Nap-CP-PEG; F) Pyr-CP-PEG; G) Ada-CP-PEG.

Table S4. Length summary of different hydrophobic nanotubes calculated from SLS data over a range of concentration gradients from 1 – 5 mg mL^{-1}.

Concentration/ mg mL^{-1}	CH_3-CP-PEG			Cyclohex-CP-PEG			Hex-CP-PEG		
	M_a	N_{agg}	Length/ nm	M_a	N_{agg}	Length/ nm	M_a	N_{agg}	Length/ nm
3.0	2.2E+06	360	169	3.8E+06	595	280	6.5E+06	1010	475
2.5	1.8E+06	291	137	3.9E+06	613	288	6.4E+06	1007	473
2.0	2.1E+06	346	163	4.1E+06	637	299	5.8E+06	901	423
1.5	1.9E+06	309	145	4.0E+06	617	290	6.6E+06	1036	487
1.0	1.5E+06	239	112	4.3E+06	672	316	6.0E+06	931	438

Concentration/ mg mL^{-1}	Nap-CP-PEG			Pyr-CP-PEG		
	M_a	N_{agg}	Length/ nm	M_a	N_{agg}	Length/ nm
3.0	1.22E+07	1944	914	1.9E+07	2948	1386
2.0	1.36E+07	2167	1018	2.3E+07	3575	1680
1.0	1.12E+07	1785	839	2.5E+07	3736	1756

Concentration/ mg mL^{-1}	Phe-CP-PEG			Ada-CP-PEG		
	M_a	N_{agg}	Length/ nm	M_a	N_{agg}	Length/ nm
2.0	4.6E+06	709	333	8.4E+06	1299	611
1.0	5.0E+06	782	368	1.1E+07	1723	810
0.5	4.7E+06	739	347	8.9E+06	1378	648

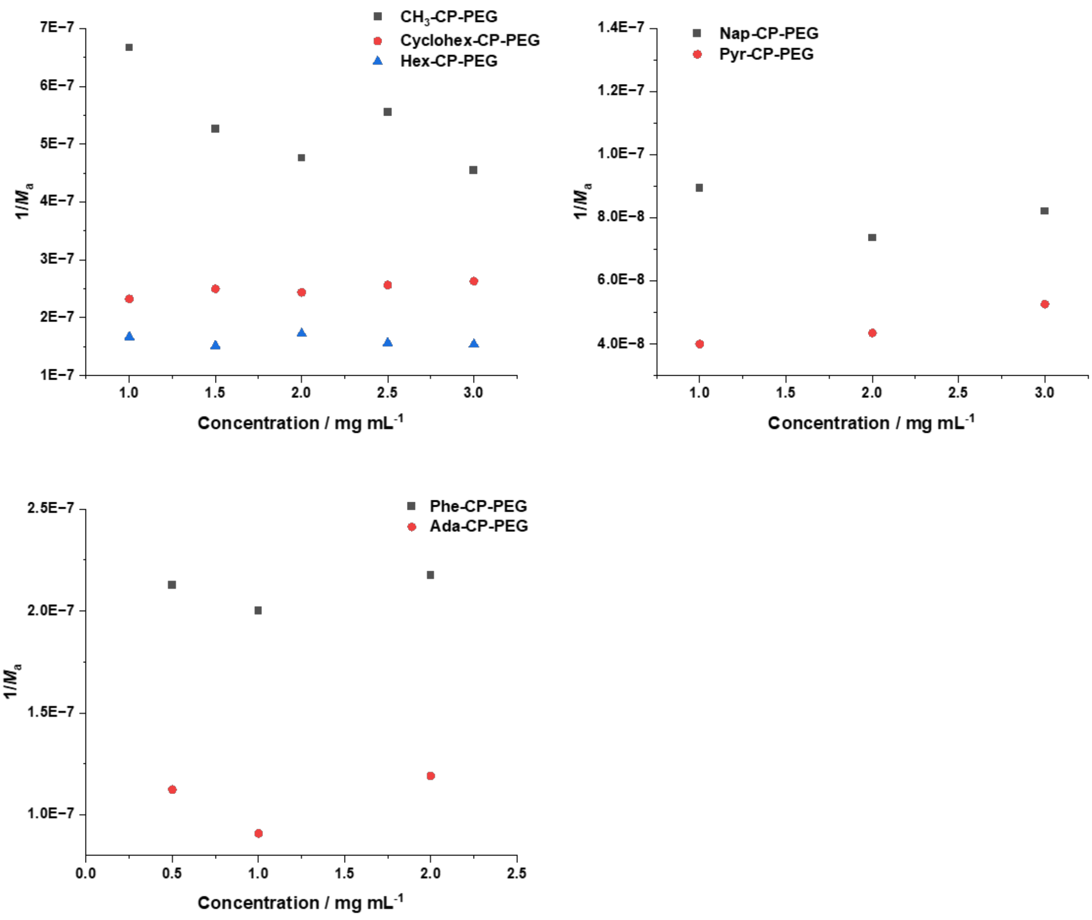

Figure S8. Evolution of $1/M_a$ of HM-CP-PEGs as a function of concentration obtained by static light scattering.

Table S5. Summary of extrapolated $\boldsymbol{M_a}$ of different nanotubes assembled in aqueous solutions through plotting the graph associated with $1/M_a$ and concentration.

Sample	Intercept ($1/M_a$)	M_a	N_{agg}*	Length /nm
CH_3-CP-PEG	6.9E-07	1.4E+06	234±52	110
Phe-CP-PEG	2.0E-07	4.9E+06	787±37	370
Cyclohex-CP-PEG	2.2E-07	4.5E+06	722±46	339
Hex-CP-PEG	1.7E-07	5.9E+06	952±53	447
Nap-CP-PEG	8.9E-08	1.1E+07	1789±179	841
Pyr-CP-PEG	3.3E-08	3.1E+07	4811±774	2261
Ada-CP-PEG	9.8E-08	1.0E+07	1619±199	761

N_{agg}*: The aggregation number was calculated from extrapolated M_a at 'zero-angle'. The error bar of each conjugate was measured by analysing all N_{agg} at different concentration conditions.

References

1. Song, Q., et al., *Efficient Artificial Light-Harvesting System Based on Supramolecular Peptide Nanotubes in Water.* Journal of the American Chemical Society, 2021. **143**(1): p. 382-389.
